# Supplementary material for: Taxonomic revision of the tarantula genus Aphonopelma Pocock, 1901 (Araneae, Mygalomorphae, Theraphosidae) within the United States
Source: Zookeys. 2016 Feb 4;(560):1–340. doi: 10.3897/zookeys.560.6264 (PMC4768370; doi:10.3897/zookeys.560.6264)
Supplement: Supplementary material 2 — Compressed files (.zip) containing Aphonopelma boxplot and PCA morphospace data. [file zookeys-560-001-s002.zip › Supp2b.zip!/Appendix 2B/Boxplot_morphospace/Aphonopelma_boxplots_males/Pygmy/T1T3_ratio_males.pdf]

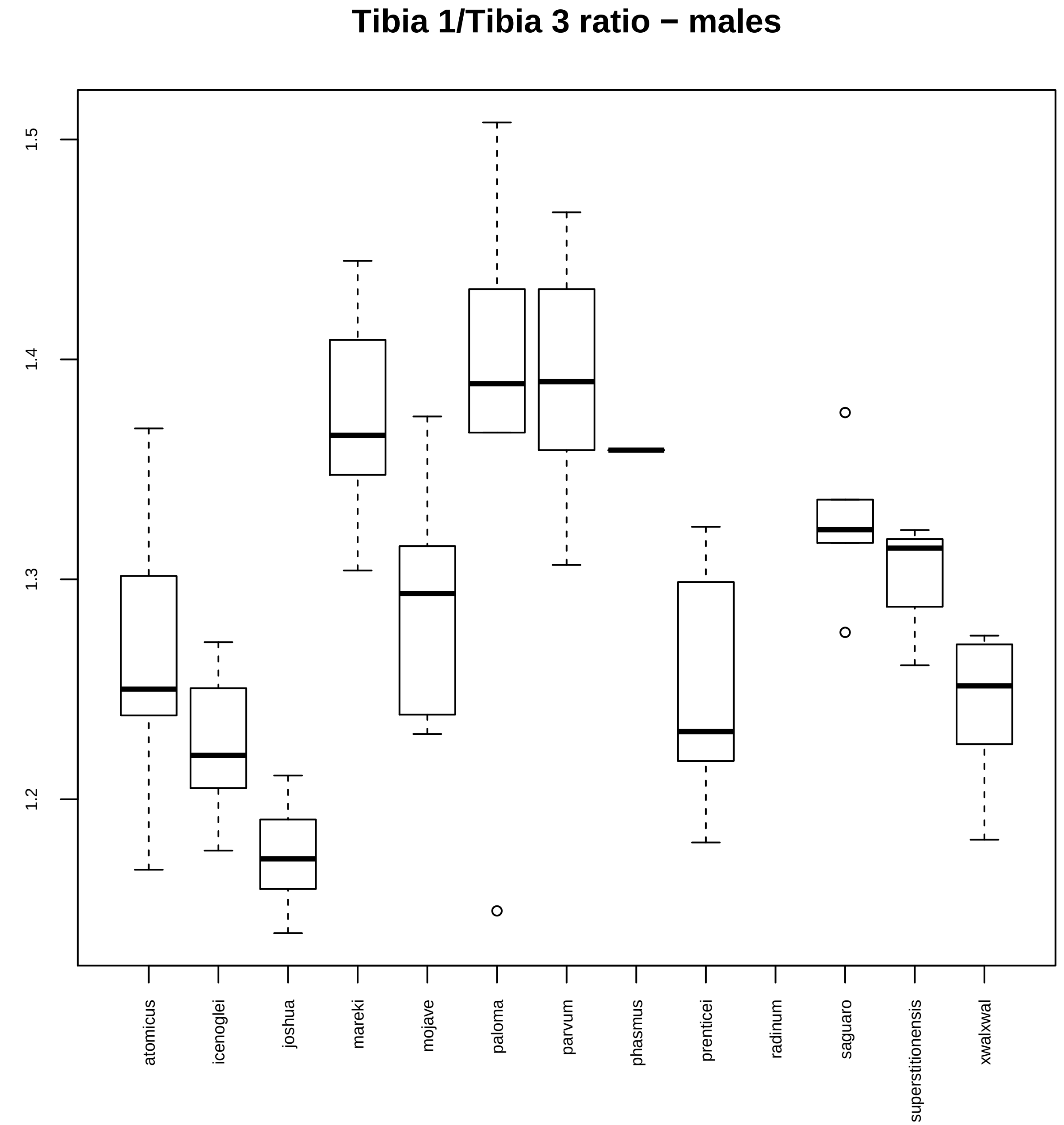
Tibia 1/Tibia 3 ratio − males
1.2
1.3
1.4
1.5
atomicus
icenoglei
joshua
mareki
mojave
paloma
parvum
phasmus
prenticei
radinum
saguaro
superstitionensis
xwalxwal
